# Ticagrelor Exerts Immune-Modulatory Effect by Attenuating Neutrophil Extracellular Traps

**DOI:** 10.3390/ijms21103625

**Published:** 2020-05-21

**Authors:** Alexandros Mitsios, Akrivi Chrysanthopoulou, Athanasios Arampatzioglou, Iliana Angelidou, Veroniki Vidali, Konstantinos Ritis, Panagiotis Skendros, Dimitrios Stakos

**Affiliations:** 1 Laboratory of Molecular Hematology, Department of Medicine, Democritus University of Thrace, 68100 Alexandroupolis, Greece; alexmitsios@gmail.com (A.M.); akrivika@hotmail.com (A.C.); thanos.arampatzioglou@gmail.com (A.A.); iliana_angelidou@yahoo.gr (I.A.); kritis@med.duth.gr (K.R.); dstakos@med.duth.gr (D.S.); 2 Natural Products Synthesis and Bioorganic Chemistry Laboratory, Institute of Nanoscience and Nanotechnology, NCSR “Demokritos”, 15310 Athens, Greece; v.vidali@inn.demokritos.gr; 3 First Department of Internal Medicine, University Hospital of Alexandroupolis, Democritus University of Thrace, 68100 Alexandroupolis, Greece; 4 Cardiology Department, University Hospital of Alexandroupolis, Democritus University of Thrace, 68100 Alexandroupolis, Greece

**Keywords:** neutrophil, neutrophil extracellular traps, inorganic polyphosphate, thrombo-inflammation, ticagrelor, myocardial infraction

## Abstract

Neutrophils through the release of neutrophil extracellular traps (NETs) containing active tissue factor (TF) are key components of thrombo-inflammation. Platelets-neutrophils interplay in ST elevation myocardial infarction (STEMI) promotes NET formation via inorganic polyphosphates (polyP) released by thrombin-activated platelets. NETs, however, are also induced by biomaterials in a platelet-independent manner. Considering the possible pleiotropic effects of Ticagrelor beyond platelet inhibition and the clinical need for novel antithrombotic strategies targeting inflammation, we investigated the effects of Ticagrelor on polyP and stent-induced NETs in STEMI. Neutrophils from healthy individuals and patients receiving Ticagrelor were stimulated with polyP or drug-eluting stents (DES) to produce NETs. To induce TF expression, neutrophils were further incubated with plasma obtained from the infarct-related artery (IRA) of STEMI patients. The effects of Ticagrelor on NETs and TF loading were assessed using fluorescence microscopy, flow cytometry, myeloperoxidase(MPO)/DNA complex ELISA, and a Western blot. Ticagrelor interrupts platelet–neutrophil interaction by attenuating NETs induced by polyP. However, Ticagrelor does not affect polyP secretion from thrombin-activated platelets. Similarly, the intracellular production of TF in neutrophils triggered by IRA plasma is not hindered by Ticagrelor. Furthermore, DES induce NETs and synchronous stimulation with IRA plasma leads to the formation of thrombogenic TF-bearing NETs. Ticagrelor inhibits stent-induced NET release. These findings suggest a novel immune-modulatory effect of Ticagrelor when it attenuates the formation of thrombogenic NETs.

## 1. Introduction

According to current knowledge, the interaction between platelets and neutrophils is crucially involved in the thrombotic process [1,2,3]. We have previously demonstrated that, in ST elevation myocardial infarction (STEMI), the interaction between platelets and neutrophils is accomplished through the secretion of linear inorganic short-chain polyphosphate (polyP) from thrombin-activated platelets [4]. PolyP is released from platelets upon their activation and can elicit potent proinflammatory responses [5,6,7]. In the microenvironment of infarct-related artery (IRA), platelets are primed to release polyP triggering neutrophils to form thrombogenic/tissue factor (TF)-bearing neutrophil extracellular traps (NETs). Prior to its expression on NETs, TF was accumulated in the cytoplasm of neutrophils, while the NET chromatin scaffold served as the vehicle for the local delivery of TF in the vicinity of the ruptured plaque [8]. Reducing the burden of cardiovascular diseases is a clinical need today and novel or alternative therapeutic strategies, which target thrombo-inflammation such as a polyP-neutrophil interaction or thrombogenic NETs, were missing until now. 

Apart from platelet-neutrophil interaction, neutrophils can react and be activated by a variety of stimuli. Biomaterials have been proposed to prime neutrophils releasing NETs that contain functional neutrophilic proteins [9,10,11]. Newer-generation drug-eluting stent (DES) has been considered a breakthrough to interventional cardiology and has now become the mainstream therapy of coronary artery stenosis. In-stent restenosis (ISR), however, remains a challenging clinical problem for the interventional cardiologists even in the advent of DES [12,13,14]. In the same context, although neutrophils have been suggested to be activated by stents, the exact mechanism remains elusive.

Inhibitors of the platelet P2Y12 receptors represent the cornerstone of the treatment strategy in acute coronary syndromes. Newer P2Y12 inhibitors, such as Ticagrelor, exhibit potent anti-platelet activity [15], but further attenuate several platelet-dependent immune reactions [16,17]. Ticagrelor has also been reported to exert platelet-independent immune-modulatory effects [18,19], but these roles are yet to be fully elucidated.

To sum up, polyP-induced NETs have a significant role in STEMI pathophysiology and, therefore, the development of polyP inhibitors emerges as a therapeutic need. In this view and taking into consideration that Ticagrelor could play an immunomodulatory role in several conditions, we studied its role on polyP-driven thrombosis in STEMI. Our data suggest that Ticagrelor is the first described safe and available human anti–polyP pharmaceutical agent. Ticagrelor attenuates the release of thrombogenic NETs triggered by both platelet-derived polyP and synthetic polyP. Moreover, the interaction between DES and neutrophils results in NET formation that can be blocked by Ticagrelor. These findings expand our currently existing knowledge on the interaction between inflammation and thrombosis, and further suggest possible pleiotropic effects of Ticagrelor beyond its known anti-platelet activity.

## 2. Results

### 2.1. Ticagrelor Attenuates polyP-Induced NETs without Affecting polyP Secretion from Platelets

**Figure 1:**
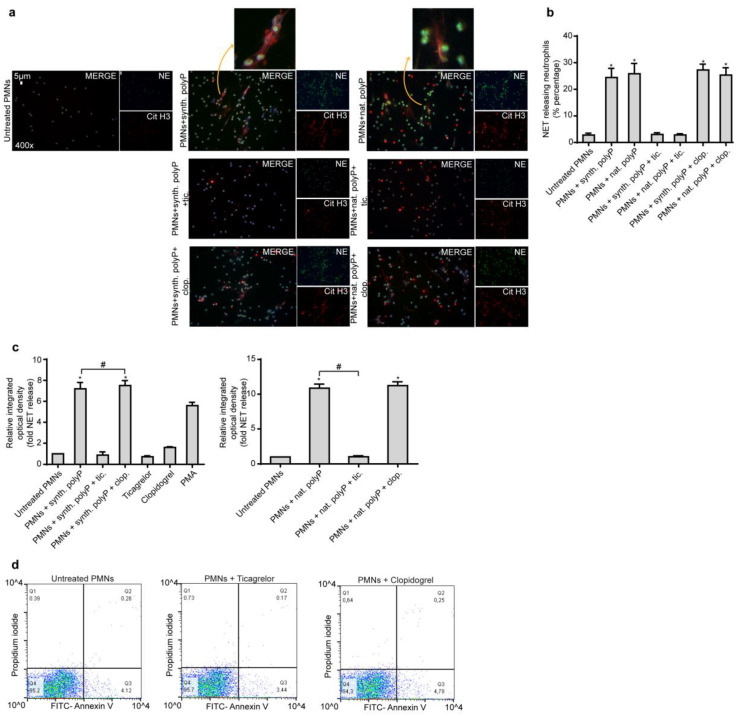
Ticagrelor inhibits NET release induced by polyP in vitro. (**a**). Fluorescence microscopy for cit-H3/NE staining in control neutrophils treated with synthetic or platelet-derived (natural) polyP, with or without pre-treatment with Ticagrelor or Clopidogrel. One representative out of six independent experiments is shown. Original magnification: 400×, Scale bar: 5μm. Blue: DAPI, Green: NE, Red: cit-H3. (**b**). Percentage of NET-releasing neutrophils as assessed by immunofluorescence. (**c**). MPO-DNA complex levels in NET structures from these stimulations, as assessed by ELISA. Relative integrated optical density was calculated compared to control NETs value. Data from six independent experiments presented as mean ± SD. Statistical significance *, # *p* < 0.05. All conditions were compared to untreated/control condition and statistical significance is indicated by the symbol “*”. Any further statistical significance of other comparisons is indicated by the symbol “#”. (**d**). Annexin V/Propidium Iodide flow cytometry of control neutrophils in the presence or absence of Ticagrelor/Clopidogrel. One representative out of six independent experiments is shown. Polymorphonuclear neutrophils (PMNs).

PolyP exerts proinflammatory and procoagulant activity [5,6,7] and short chain polyP released by activated platelet is able to prime neutrophils to undergo NET formation. Due to the fact that NETs deliver thrombogenic signals during thrombus propagation and stabilization, first, we assessed the effect of Ticagrelor and Clopidogrel on polyP-induced NET release. We used synthetic polyP to induce NETs on healthy human neutrophils and healthy human neutrophils pre-treated with Ticagrelor or Clopidogrel. Since Clopidogrel is a pro-drug that requires activation in the liver, we used a bioactive isomer of the drug. We found that, compared to Clopidogrel, Ticagrelor significantly attenuated the formation of polyP-induced NETs from human neutrophils in vitro (Immunofluorescence, MPO/DNA complex ELISA) (Figure 1a–c). Similarly, to synthetic polyP, Ticagrelor significantly attenuated the NETotic effect of natural polyP derived from thrombin-activated platelets, compared to Clopidogrel (Immunofluorescence, MPO/DNA ELISA) (Figure 1a–c). Lastly, Ticagrelor does not affect the viability of neutrophils by inducing apoptosis or necrosis as assessed by Annexin V/Propidium Iodide (PI) flow cytometry analysis (Figure 1d).

**Figure 2:**
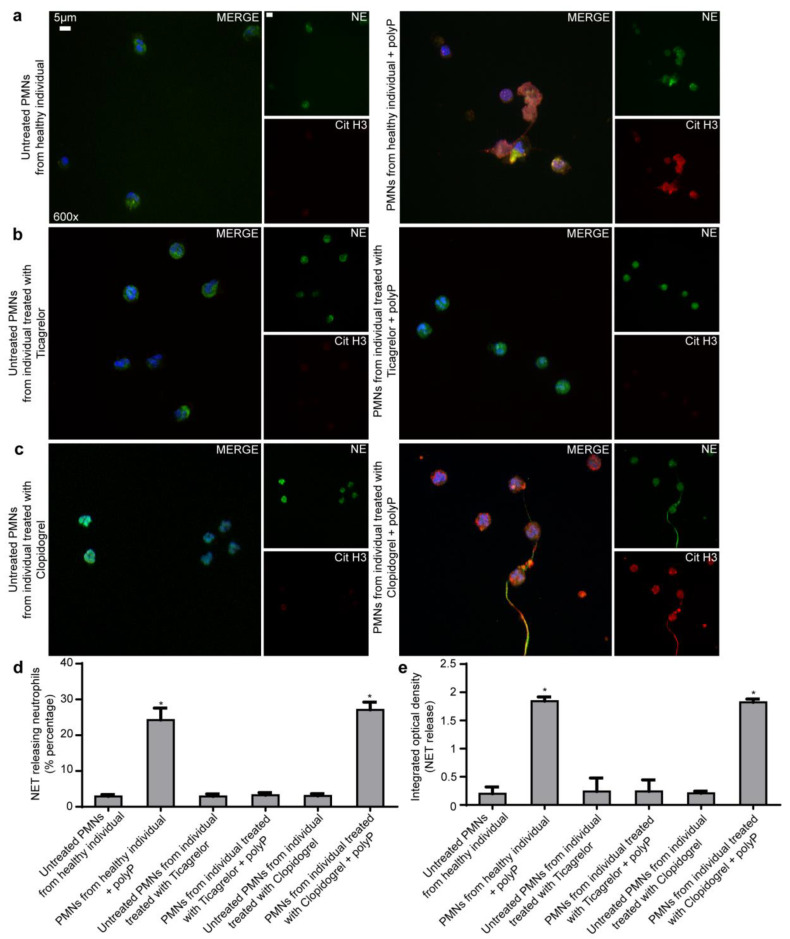
Neutrophils from individuals receiving Ticagrelor were more resistant to NETotic stimulation from polyP. (**a**–**c**). Fluorescence microscopy for cit-H3/NE staining in neutrophils isolated from a patient with a previous acute coronary syndrome and stent placement that receives Ticagrelor or Clopidogrel as a main antiplatelet treatment and neutrophils from a healthy individual, with or without synthetic polyP. One representative out of five independent experiments is shown. Original magnification: 600×, Scale bar: 5 μm. Blue: DAPI, Green: NE, Red: cit-H3. (**d**). Percentage of NET-releasing neutrophils as assessed by immunofluorescence. (**e**). MPO-DNA complex levels in NET structures from these stimulations, as assessed by ELISA. Data from five independent experiments presented as mean ± SD. Statistical significance * *p* < 0.05. All conditions were compared to untreated/control condition and statistical significance is indicated by the symbol “*”. Polymorphonuclear neutrophils (PMNs).

In order to further strengthen our in vitro findings, we performed stimulation experiments in neutrophils obtained from coronary artery disease (CAD) patients receiving Ticagrelor or Clopidogrel and from healthy individuals (controls). The basal levels of NETs in CAD patients were low and comparable to that of controls (Figure 2e). Ticagrelor-treated CAD-patients-derived neutrophils were more resistant to NETotic stimulation from polyP when compared to control neutrophils under similar polyP doses. This suggests that Ticagrelor exerts anti-thrombo-inflammatory effects by attenuating NETs (Figure 2a,b,d,e). On the other hand, Clopidogrel-treated CAD-patients-derived neutrophils do not have diminished NET release (Figure 2a,c–e). The formation of NETs was evaluated by Immunofluorescence, MPO/DNA ELISA. 

**Figure 3:**
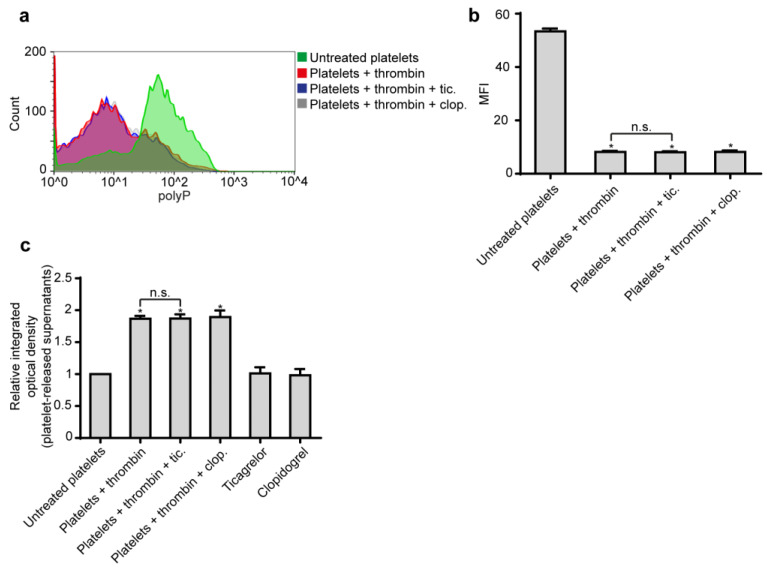
Ticagrelor does not inhibit polyP release from platelets. (**a**). Representative flow cytometry analysis and (**b**). relative mean fluorescent intensity (MFI) of polyP on control platelets treated with thrombin, with or without pre-treatment with Ticagrelor or Clopidogrel. MFI—mean fluorescence intensity. (**c**). Quantification of the released polyP with JC-D8 polyP-specific fluorescent probe. Relative I integrated optical density OD was calculated compared to control platelets value. (**a**). One representative out of six independent experiments is shown. (**b**,**c**) Data from six independent experiments presented as mean ± SD. Statistical significance * *p* < 0.05. n.s.: non-significant. All conditions were compared to an untreated/control condition and statistical significance is indicated by the symbol “*”. Any further non-statistical significance of other comparisons is indicated by the symbol “n.s.”.

Since Ticagrelor inhibited the formation of NETs induced by polyP and considering that polyP is the major mediator of platelet-induced NETosis, we next investigated the role of Ticagrelor in polyP secretion from platelets. We found that Ticagrelor and Clopidogrel do not affect polyP release from thrombin-activated platelets, as assessed by flow cytometry and fluorometry (Figure 3). 

The results suggest that, beyond its antiplatelet effects, Ticagrelor exerts direct immune-regulatory properties on neutrophils without affecting polyP release from platelets.

### 2.2. Ticagrelor Effect on Neutrophils Does not Rely on P2Y12 Receptor and Autophagy

**Figure 4:**
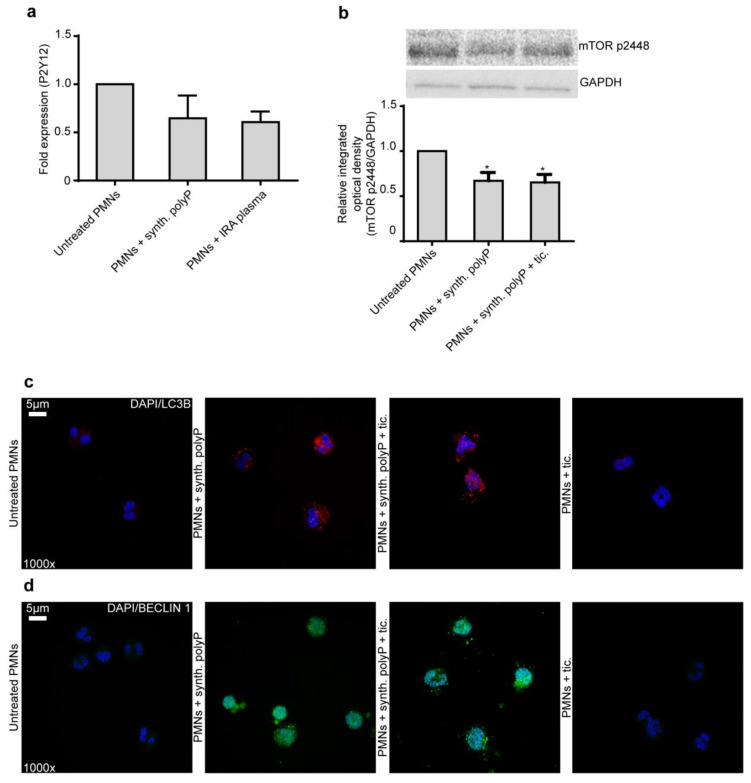
Ticagrelor does not signal through the P2Y12 or the autophagy pathway in neutrophil. (**a**). mRNA fold expression for P2Y12 receptor in control neutrophils treated with synthetic polyP and IRA plasma. Data from three independent experiments presented as mean ± SD. There is no statistical significance. (**b**). Western blot of mTOR p2448 in control neutrophils treated with synthetic polyP, with or without pre-treatment with Ticagrelor. One representative out of four independent experiments is shown. Relative integrated optical density was calculated compared to untreated PMNs value. Data from four independent experiments presented as mean ± SD. Statistical significance * *p* < 0.05. All conditions were compared to untreated/control conditions and statistical significance is indicated by the symbol “*”. (**c**,**d**). Confocal microscopy for DAPI/LC3B or DAPI/BECLIN 1 staining in control neutrophils treated with synthetic polyP, with or without pre-treatment with Ticagrelor. One representative out of six independent experiments is shown. Original magnification: 1000×, Scale bar: 5 μm. Blue: DAPI. Red: LC3B. Green: BECLIN 1. Polymorphonuclear neutrophils (PMNs).

We sought to investigate signaling pathways related to the action of Ticagrelor and NET formation, such as the P2Y12 receptor and the autophagy pathway, respectively. Based on the above and other previous observations that Ticagrelor affects immunity and neutrophils [20,21], we examined whether the P2Y12 receptor is expressed by neutrophils by using qRT-PCR. We also examined whether polyP or IRA plasma could have an effect on this expression. The qRT-PCR led to a non-specific product (high *C*t value), showing that P2Y12 is not expressed by neutrophils (Figure 4a). This finding is in accordance with a previous study showing that, by using a Western blot, neutrophils do not express P2Y12 receptors [22]. These results suggest that the effects of Ticagrelor on neutrophil function are not mediated by its classical target (the P2Y12 receptor) as its effects on platelets.

We have shown previously that polyP is able to cause de-phosphorylation of mammalian target of rapamycin (mTOR), which results in the induction of autophagy and, subsequently, in the release of NETs by neutrophils [4]. Therefore, we examined whether Ticagrelor inhibits NET formation through phosphorylation of mTOR. However, we saw via Western blot that Ticagrelor cannot restore the de-phosphorylation caused by synthetic polyP (Figure 4b). In addition, we confirmed our result via immunofluorescence for LC3B and Beclin-1. Stimulation of healthy neutrophils with polyP resulted in increased autophagy while Ticagrelor is not able to inhibit autophagy induction (Figure 4c,d).

### 2.3. Ticagrelor Has no Effect on Intracellular Expression of TF

**Figure 5:**
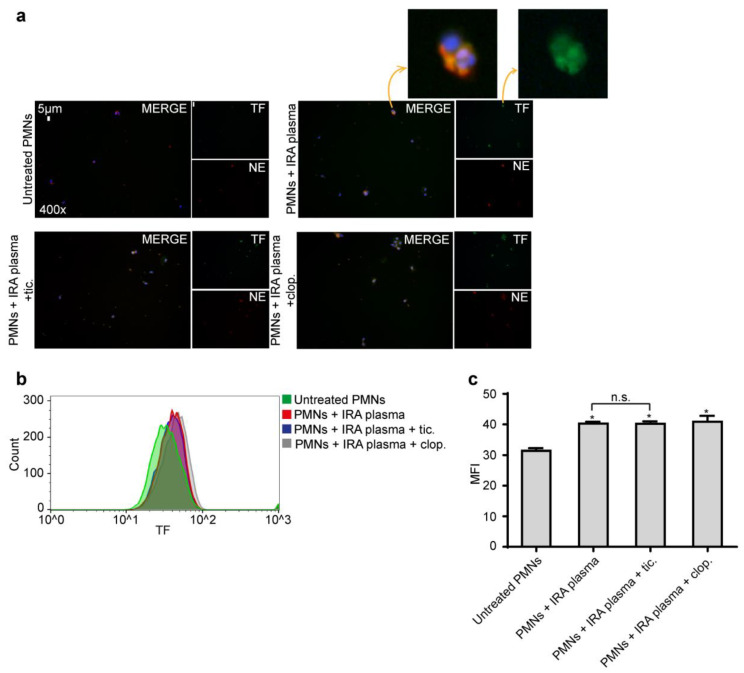
Τicagrelor is not able to inhibit intracellular expression of tissue factor (TF). (**a**). Fluorescence microscopy for TF/NE staining in control neutrophils treated with IRA plasma, with or without pre-treatment with Ticagrelor or Clopidogrel. One representative out of six independent experiments is shown. Original magnification: 400×, Scale bar: 5 μm. Blue: DAPI, Green: TF, Red: NE. (**b**). Representative fluorescence-activated cell-sorting analysis and (**c**) relative MFIs of TF in control neutrophils treated with IRA plasma, with or without pre-treatment with Ticagrelor or Clopidogrel. Data from six independent experiments presented as mean ± SD. Statistical significance * *p* < 0.05. n.s.: non-significant. All conditions were compared to the untreated/control condition and statistical significance is indicated by the symbol “*”. Any further non-statistical significance of other comparisons is indicated by the symbol “n.s.”. MFI—mean fluorescence intensity. (**a**,**b**) One representative out of six independent experiments is shown. Polymorphonuclear neutrophils (PMNs).

We have previously shown that thrombosis-related inflammatory stimuli (IRA-derived plasma) induce intracellular accumulation of TF in neutrophils [8]. Therefore, in order to investigate the effects of Ticagrelor on the expression of TF in neutrophils, neutrophils were cultured in the presence of IRA-derived plasma. None of the drugs was able to inhibit TF accumulation on neutrophils, as assessed by immunofluorescence and flow cytometry (Figure 5). However, because NETs function as a scaffold for the extracellular exposition of functional TF inhibition, NET formation by Ticagrelor can block the activity of neutrophil-derived TF.

### 2.4. Ticagrelor Inhibits Thrombotic NET Release Induced by Drug Eluting Coronary Stents

**Figure 6:**
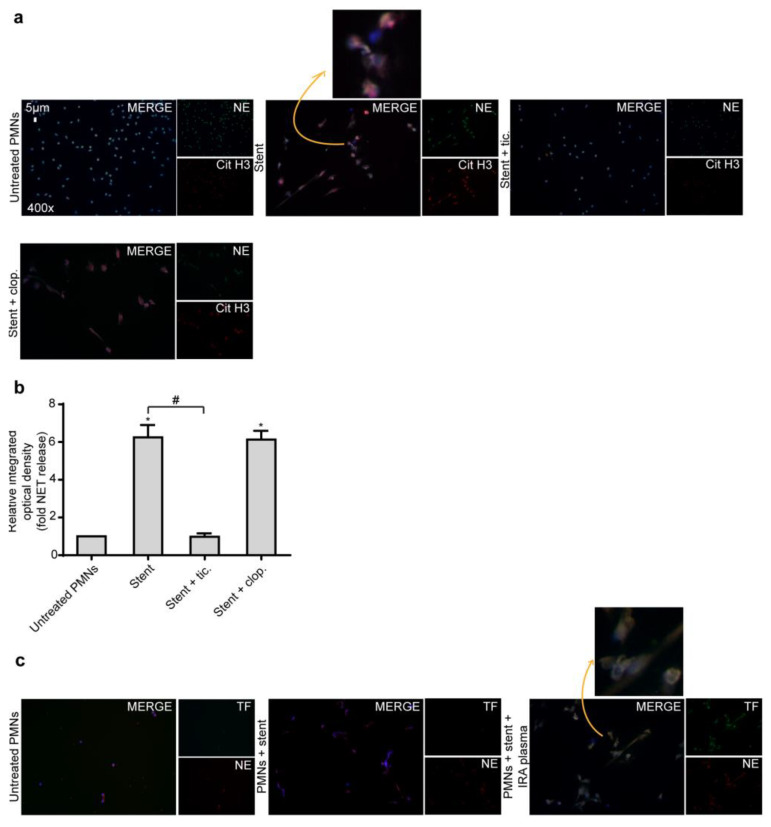
Coronary stents induce NET formation. (**a**). Fluorescence microscopy for cit-H3/NE staining in control neutrophils treated with stent and with or without pre-treatment with Ticagrelor or Clopidogrel. One representative out of six independent experiments is shown. Original magnification: 400×, Scale bar: 5 μm. Blue: DAPI, Green: NE, Red: cit-H3. (**b**). MPO-DNA complex levels in NET structures from control neutrophils treated with stent, with or without pre-treatment with Ticagrelor or Clopidogrel, as assessed by ELISA. Relative integrated optical density was calculated when compared to control NETs value. Data from six independent experiments presented as mean ± SD. Statistical significance *, # *p* < 0.05. All conditions were compared to untreated/control conditions and statistical significance is indicated by the symbol “*”. Any further statistical significance of other comparisons is indicated by the symbol “#”. (**c**). Fluorescence microscopy for TF/NE staining in control neutrophils treated with stent and IRA plasma. One representative out of six independent experiments is shown. Original magnification: 400×, Scale bar: 5 μm. Blue: DAPI, Green: TF, Red: NE. Polymorphonuclear neutrophils (PMNs).

Although significantly reduced by newer third generation stents, stent restenosis and thrombosis still represent the Achilles’ heel of coronary interventions. Since NETs are a significant component of thrombotic material obtained from patients with stent thrombosis [8,23], we studied the ability of coronary stents to induce NET formation in vitro. Thus, neutrophils were incubated with DES and NET generation was assessed by MPO/DNA complex ELISA and immunofluorescence. Following three hours of co-incubation, DES induced the generation of NETs from healthy neutrophils. Based on the above, we wished next to investigate the effects of Ticagrelor on NETs induced by coronary stents. We found that, compared to Clopidogrel, Ticagrelor significantly attenuated the NETotic effect of coronary stents in vitro, as determined by immunofluorescence and MPO/DNA ELISA (Figure 6a,b).

Furthermore, co-stimulation of neutrophils with IRA-derived plasma and stents resulted in the formation of TF-loaded NETs (Figure 6c). Collectively, these results suggest that the interaction of neutrophils with coronary stents, in a STEMI environment, generates thrombogenic NETs.

Taken together, our results suggest that Ticagrelor exerts favorable effects on immunity and inflammation by inhibiting the formation of thrombogenic NETs in vitro induced by either platelet-neutrophil interaction (through polyP secretion) or coronary stents.

## 3. Discussion

The previously mentioned data suggest the particular activity of Ticagrelor to inhibit the formation of NETs by neutrophils triggered by polyP (both synthetic and platelet-derived) and coronary stents. This immune-modulatory property of Ticagrelor observed in human neutrophil cultures in vitro is independent of its antiplatelet activity and, thus, precludes any indirect effect of the drug on NETs by inhibiting platelet activation. This dual inhibitory potential of Ticagrelor (on platelets and neutrophils/NETs) is particularly important since platelet-neutrophil interaction is a perquisite step for NET generation and the subsequent thrombotic events [1,2,3,24]. When confirming these in vitro observations, we found a significantly attenuated NETotic activity of neutrophils derived from patients receiving Ticagrelor following stimulation with polyP.

It is well established that neutrophils promote thrombotic manifestations through NET release [8,25,26,27,28,29]. Accordingly, regulating exaggerated neutrophil activity and NET formation may hold therapeutic implications in several inflammatory, autoimmune, and thrombotic diseases [4,30,31].

Ticagrelor is an orally administrated reversible inhibitor directly targeting the adenosine diphosphate receptor P2Y12. The drug has a rapid onset of action and pronounced platelet inhibition. When administrated to patients with acute coronary syndromes, it appears to reduce the rate of deaths from cardiovascular causes, myocardial infarction, or stroke [32]. The reduction in all-cause mortality with Ticagrelor (4.5%) vs. Clopidogrel (5.9%) was significant (HR 0.78, 95% CI 0.69–0.89, *p* < 0.001) and lower based on previous studies raising questions about pleiotropic effects of the drug beyond its antiplatelet activity [18]. Moreover, participants in the Ticagrelor group of the PLATO study showed significantly lower inflammatory markers of neutrophil activation during sepsis when compared to the Clopidogrel group [33]. In line with this, Ticagrelor was found to reduce thrombo-inflammatory markers in pneumonia patients during the XANTHIPPE study [34]. Lastly, Ticagrelor prevented neutropenia induced by intravenous injection of Escherichia coli endotoxin in healthy volunteers [19]. Since NETs represent a form of cell death induced by gram (-) sepsis [35], the above observation may similarly suggest a possible inhibitory effect of Ticagrelor on NET formation. Considering all the above, the proposed inhibition of NET formation by Ticagrelor seems not to be associated with increased risk of infection, according to current experimental findings that suggest a direct antimicrobial effect of Ticagrelor [36].

It was recently found that Ticagrelor induces alterations in thrombus ultra-structure and modifies clot stability, which reduces resistance to fibrinolysis in an experimental human sepsis model [19]. It has been previously shown that the presence of extracellular histones, DNA, and NETs enhance thrombus stability and resistance to thrombolysis [37,38], while residual thrombotic material obtained from STEMI patients with spontaneous thrombus resolution is characterized by the absence of NETs [8]. According to our results, it is tempting to hypothesize that Ticagrelor may confer favorable effects on thrombus mechanical properties by manipulating NET release during thrombus formation. 

Intracellular TF is in an inactive coagulant state when it requires activation to reach its full potency [39]. One of the mechanisms that have been described for neutrophil TF activation is its exposure on NETs [4,8,40]. Upon NET generation, neutrophil-derived TF is functional and able to induce PAR-1 mediated platelet activation and thrombin generation [8,41]. Since NETs are required for TF functioning, inhibition of NET formation by Ticagrelor can prevent its activation. Nevertheless, a strategic treatment that combines Ticagrelor, which blocks the release of NETs, with another agent that is able to inhibit the intracellular expression of TF [4,9,41] could obtain better results in clinical practice.

Furthermore, the inhibition of NET generation triggered by coronary stents is also important since a recently published study showed that NETs represent a hallmark in thrombus specimens obtained from patients with stent thrombosis, which supports their pathophysiological relevance [23].

Until today, there were no therapeutic agents targeting polyP despite the fact that it is the major mediator of platelet-induced NETosis and that NETs have a crucial role in thrombo-inflammation. Ιn a recent study conducted by our group, it was demonstrated that polyP is able to induce NET release via de-phosphorylation of mTOR and autophagy activation [4]. Several studies indicate that autophagy is possibly a mechanism linked to NET formation [42,43,44,45]. Moreover, we have previously suggested that the cytokine IL-29 is a potent natural inhibitor of polyP-induced NET release [4]. Ticagrelor is an antiplatelet drug already widely used in clinical practice. It seems to have NET inhibitory effect since IL-29 does not act through the mTOR/autophagy pathway. Ticagrelor is the first human pharmaceutical agent in use, which also has potent anti-polyP activity. In the present study, we have not elucidated the mechanism of this novel action of Ticagrelor on neutrophils, but it highlighted the immune-modulatory effect of the drug by attenuating thrombotic NETs. Moreover, the fact that Ticagrelor is able to inhibit NET release, which is caused either by polyP or coronary stents, suggests that common mediators are likely involved in this effect of Ticagrelor or it might act at multiple, interconnected pathways that lead to NET formation [4]. 

Several studies in platelets indicated that Ticagrelor inhibits metabolic clearance of adenosine by increasing its levels [46]. Adenosine activates adenylate cyclase, forming cyclic adenosine monophosphate, which has been found to inhibit NET formation likely through ROS reduction [47,48]. Thus, Ticagrelor could possibly attenuate NET release independently from the P2Y12 receptor by inhibiting cAMP.

Some biomaterials such as hemodialysis filters can cause the over-expression of TF by neutrophils, enhancing, in this way, neutrophil-driven thrombotic manifestations [9]. In STEMI, coronary stents are able to induce TF-decorated NETs only via a two-“hit” process [31] and not by themselves. The first “hit” is the inflammatory environment that primes neutrophils to express TF. The second “hit” is the stent-neutrophil interaction required for the induction of NET formation. Since some biomaterials induce the expression of cytokines/proteins by neutrophils, the improvement of their biocompatibility is crucial.

In conclusion, the present study suggests a direct immune-modulatory effect of Ticagrelor by inhibiting NET release from neutrophils triggered by polyP or coronary stents. Our in vitro results on NET release have also been confirmed in ex vivo samples from patients receiving Ticagrelor or Clopidogrel. Although it would have been of interest to assess our hypothesis on stent-induced NET release by using ex vivo experiments, this is difficult to address in patients who receive stents since this approach would require control groups and repeated interventions for multiple samplings. This limitation can be overcome by using experimental animal models in future studies. Further experimental efforts as well as additional in vivo and ex vivo studies are required to elucidate the exact mechanisms that underly this inhibitory action of Ticagrelor, which may have clinical significance by proposing novel therapeutic targets.

## 4. Material and Methods

### 4.1. Patients and Sample Collection

Based on our previous in vitro data showing that Ticagrelor (unlike Clopidogrel) inhibited NET formation from neutrophils, we sought to investigate the potential that Ticagrelor is able to inhibit NET release in CAD patients (ex vivo). For this reason, peripheral blood neutrophils were isolated from 10 patients with stent placement due to previous acute coronary syndrome receiving Ticagrelor (*n* = 5) or Clopidogrel (*n* = 5) as a main antiplatelet treatment for at least 6 months. None of the patients suffered from diabetes mellitus, or chronic inflammatory disease or malignancy. To ensure accuracy of the results, aspirin was discontinued 7 days before sampling as allowed by current focused update on Dual Antiplatelet Therapy Guidelines of the European Society of Cardiology [49,50]. Peripheral blood neutrophils and platelets were also obtained from 10 age-matched and gender-matched healthy individuals (control) as previously described [4]. Characteristics of coronary artery disease patients and healthy individuals of the study are presented in Table S1.

For the in vitro stimulation experiments, thrombus material and surrounding blood was aspirated by using a dedicated aspiration catheter from the IRA of patients with STEMI during primary percutaneous coronary intervention (PCI). The methods used for selective blood sampling and handling to obtain IRA-derived plasma have been previously described [8]. 

Written informed consent was obtained from each individual. The study protocol design was in accordance with the Declaration of Helsinki and was approved by the Ethics Review Board of the University Hospital of Alexandroupolis.

### 4.2. Stimulation and Inhibition Studies

In vitro stimulations and inhibitions of neutrophils and platelets were performed as previously described [4]. In vitro stimulations and inhibitions of neutrophils from control individuals were performed in Roswell Park Memorial Institute (RPMI) medium. To induce NET release, 1.5 × 10^6^ neutrophils were treated with chemically synthetized short chain polyP (approximately 75 phosphates, 2 μg/mL, Kerafast Inc, Boston, MA, USA) or natural polyP released from thrombin-activated control platelets (2 μg/mL) for 3 h at 37 °C. Neutrophils were also incubated with phorbol 12-myristate 13-acetate (PMA) (40 ng/mL, Sigma-Aldrich, St Louis, MO, USA), which is a generic inducer of NET release for 3 h. To induce TF expression, neutrophils were treated with 5% plasma isolated from IRA blood of STEMI patients (IRA plasma), for 3 h at 37 °C. Plasma isolated from coronary blood of control individuals was used as a negative control. To examine the effect of DES, neutrophils were cultured in RPMI medium in the presence of a DES as well as 5% IRA plasma or not for 3 h at 37 °C. To generate natural polyP, 5 × 10^6^ platelets from healthy individuals were stimulated with thrombin for 90 min at 37 °C. To induce autophagy, neutrophils were treated with chemically synthetized short chain polyP for 45 min at 37 °C.

To examine whether Ticagrelor (provided by AstraZeneca for research purposes only) could inhibit polyP, DES, and/or IRA plasma effect on neutrophils, cells were pre-treated with 5 μM Ticagrelor for 30 min, prior to their exposure on each inducer. A bioactive isomer of Clopidogrel (Sigma-Aldrich, C0614, Munich, Germany) was used at a dose of 60 μΜ, as a control drug for 30 min, prior to exposure on each inducer [51,52,53,54]. Appropriate time and dose experiments were performed prior to Ticagrelor and Clopidogrel in vitro stimulations. 

### 4.3. PolyP Detection Using Fluorescent Probes

PolyP-specific fluorescent probes JC-D7 and JC-D8 were synthesized according to previous literature methods with slight modifications [4,5]. For determination of polyP using fluorometry, probes were used at a concentration of 20 μM, as described previously [4].

### 4.4. Other Experimental Procedures

Immunofluorescence staining, NET structures isolation, MPO/DNA complex ELISA, and FACS analysis were performed as previously described [2,4,55,56,57]. 

#### 4.4.1. Immunofluorescence Staining

Samples were stained using a rabbit anti-citrullinated H3 (R2+R8+R17) (1/200 dilution, Abcam, ab5103, Cambridge, UK) and a mouse anti-neutrophil elastase (NE) mAb (1/200 dilution, Santa Cruz Biotechnology Inc, sc-55548, TX, USA), or a mouse anti-TF mAb (1/200 dilution; Sekisui Diagnostics, 4509, MS, USA) and a rabbit anti-neutrophil elastase (NE) mAb (1/200 dilution, Santa Cruz Biotechnology Inc, sc-25621, TX, USA), or a rabbit anti-Microtubule-Associated Protein 1 Light Chain Beta (LC3B) polyclonal Ab (1/200 dilution, Sigma-Aldrich, L7543, Munich, Germany) or a mouse anti- BECN1 mAb (1/200 dilution, Santa Cruz Biotechnology Inc, sc-48381). A polyclonal goat anti-rabbit Alexa fluor 647 antibody (Invitrogen, A-21244, Carlsbad, CA, USA), a polyclonal rabbit anti-mouse Alexa fluor 488 antibody (Invitrogen, A-11059), and a polyclonal goat anti-rabbit Alexa fluor 594 antibody (Invitrogen, R-37117) were utilized as secondary antibodies. DNA was counterstained by using DAPI (Sigma-Aldrich, D9542). 

Cell preparations were visualized using a fluorescence microscope (OLYMPUS BX51) with an established NIKON camera (model DS-Fi1) and a confocal microscope (Spinning Disk, Andor Revolution Confocal System, Belfast, UK) in a PLAPON 606O/TIRFM-SP, NA 1.45, and UPLSAPO 100XO, NA 1.4 objectives (Olympus). The percentage of NET-releasing cells was determined by examining 200 cells in a double-blind experimental procedure.

#### 4.4.2. NET Structures Isolation

1.5 × 10^6^ neutrophils were seeded in 6-well culture plates (Corning Incorporated, New York, NY, USA) in RPMI medium. Their treatment was performed as described in the stimulation and inhibition studies section. After treatment, the medium was removed and cells were washed twice with pre-warmed RPMI medium. Additionally, 1 ml of pre-warmed RPMI was added to each well and NET structures were collected on supernatant medium after vigorous agitation and sequential centrifugation, as previously described [58].

#### 4.4.3. MPO/DNA Complex ELISA

To quantify NET release, the MPO-DNA complex was measured in NETs isolated from 1.5 × 10^6^ neutrophils, as previously described [59]. In brief, an F-bottom high-binding 96-well-plate (Corning Incorporated) was coated with human anti-MPO ab (1/500 dilution, Hycult Biotech, HM2164, Wayne, MI, USA) overnight at 4 °C. Isolated NET structures were added to the wells. Anti-double strand DNA ab (1/25 dilution, Sigma-Aldrich, 11774425001) was used as a detection antibody for 2 h with shaking at room temperature. Peroxidase substrate [2,2′-azinobis [3-ethylbenzthiazolinesulfonic acid, ABTS]] was added to the wells. After 10 min of incubation at room temperature in the dark, absorbance was measured at 405 nm. NET release was calculated as a fold increase when compared to untreated neutrophils.

#### 4.4.4. Flow Cytometry Analysis

For the analysis of apoptosis/necrosis, untreated and treated with Ticagrelor neutrophils groups were stained with Annexin V FITC (BD Biosciences, NJ, USA, 556420, 20 min at 4 °C), an apoptotic marker, and propidium iodide (PI, Sigma-Aldrich, 556463, 10 min at 4 °C), which stains necrotic cells. Analysis was performed in a FACScalibur flow cytometer (BD Biosciences).

To detect polyP, in vitro-stimulated platelets were stained using DAPI (Sigma-Aldrich, D9542). The peak emission wavelength of free DAPI is 475 nm, while the peak emission wavelength of the DAPI-polyP complex is 525 nm [57,60]. Anti-CD61-PerCP antibody (BD Biosciences) was used as a platelet specific marker. Platelets were immediately diluted in 500 μL of HEPES buffer and analysed by flow cytometry (BD Biosciences, FACScan). Analysis was performed on 10^4^ gated CD61-positive events.

To determine the intracellular expression of TF, 5 × 10^5^ neutrophils were fixed and permeabilized by Fix and Perm (Invitrogen), according to the manufacturer’s instructions, and stained with a mouse anti-human TF mAb (Sekisui Diagnostics, 4509), for 3 h at 4 °C. Cells were washed and incubated for 30 min at 4 °C with FITC-labelled goat anti-mouse Ab (BD Biosciences, 555988). FITC-labelled mouse IgG1 (BD Biosciences, 349041) was used as an isotype control.

All data were analyzed using Flowjo V10.0.0.4 (BD Biosciences, Franklin Lakes, NJ, USA).

#### 4.4.5. RNA Isolation, cDNA Synthesis, and qRT-PCR

Total RNA was extracted, using the TRIZOL reagent (Invitrogen), according to the manufacturer’s instructions. cDNA synthesis was performed using Superscript III Reverse Transcriptase kit (Invitrogen). Real-time PCR was performed using SYBR Green qPCR Master Mix (2x) gene expression master mix (Fermentas, St. Leon-Rot, Germany) on a Chromo4TM Real-Time Detector (Bio-Rad, Hercules, CA, USA). GAPDH was used as the house-keeping gene to normalize the expression levels of target genes. The following oligonucleotide primers, designed by Beacon Designer™ 4.0, were used: P2Y12 (forward: 5′-CCTCCAGAATCAACAGTTATC AG-3′, reverse: 5′-CAGGACAGTGTAGAGCAGTG-3′), GAPDH (forward: 5′-GGGAAGCTTGTCATCAATGG-3′, reverse: 5’-CATCGCCCCACTTGATTTTG-3′). The PCR protocol used included the following steps: 52 °C for 5 min, 95 °C for 2 min, 35 cycles of: 95 °C for 15 s and 51 °C for 40 s, 52 °C for 5 min, and a melting curve analysis.

#### 4.4.6. Western Blot Analysis

To study autophagy induction, a rabbit anti-human mTOR p2448 polyclonal Ab (1/500 dilution, Cell Signaling Technology) were used. To verify equal loading in cell lysates, membranes were re-probed for GAPDH (1/1000 dilution, Santa Cruz Biotechnology). The Western blot was performed as described by the manufacturers of the antibodies. Protein expression was measured using Image Lab 5.2.1 (Bio-Rad), and it is expressed as relative integrated optical density compared with untreated neutrophils (relative integrated optical density for untreated cells is 1).

### 4.5. Statistical Analysis

Statistical analyses were performed using Kruskal-Wallis test, which was followed by Dunn’s test for multiple comparisons. *p*-values less or equal to 0.05 were considered significant. All conditions were compared to untreated/control condition and statistical significance is indicated by the symbol “*”. Any further statistical significance of other comparisons is indicated by the symbol “#”. All statistical analyses were performed using GraphPad Prism 6.

### 4.6. Ethical Approval

All procedures performed in studies involving human participants were in accordance with the ethical standards of the Ethics Review Board of the University Hospital of Alexandroupolis (approval date 15 May 2017) and with the 1964 Helsinki declaration and its later amendments or comparable ethical standards. Written informed consent was obtained from each individual.

## Supplementary Materials

The following are available online at https://www.mdpi.com/1422-0067/21/10/3625/s1, Table S1: Characteristics of coronary artery disease patients and healthy individuals (controls).
