# Supplementary material for: Ticagrelor Exerts Immune-Modulatory Effect by Attenuating Neutrophil Extracellular Traps
Source: Int J Mol Sci. 2020 May 21;21(10):3625. doi: 10.3390/ijms21103625 (PMC7279443; doi:10.3390/ijms21103625)
Supplement: Supplementary file 1 [file ijms-21-03625-s001.pdf]

Table S1. Characteristics of coronary artery disease patients and healthy individuals (controls).

Parameters	Patients	Healthy Individuals
**N**	10	10
**Gender (M/F)**	8/2	8/2
**Mean age±SD**	63±3	61±4
**Time since index PCI (months)**	8±3	-
**Ticagrelor**	5	-
**Clopidogrel**	5	-
**Statins**	9/10	-
**Antihypertensives**	5/10	-

PCI: percutaneous coronary intervention.
